# Spontaneous Rupture of the Extensor Pollicis Longus in a Semi-professional Bodybuilder

**DOI:** 10.7759/cureus.35062

**Published:** 2023-02-16

**Authors:** Issam Boulazaib, Adnane Lachkar, Najib Abdeljaouad, Hicham Yacoubi

**Affiliations:** 1 Orthopedic Trauma Department, Mohammed VI University Hospital, Oujda, MAR; 2 Orthopedics, Faculty of Medicine and Pharmacy of Oujda, Mohammed First University of Oujda, Oujda, MAR

**Keywords:** bodybuilder, lister tubercle, extensor policis longus, tendon reconstruction, sport injury

## Abstract

Spontaneous rupture of the extensor pollicis longus is a rare injury. Distal radius fractures and rheumatisms are the principal causes, but there is an increase in cases related to some professional or sports activities. We report a case of a semi-professional bodybuilder who presented with a full loss of interphalangeal thumb extension and retropulsion following trivial trauma of the left wrist. Ultrasound confirmed the diagnosis of spontaneous rupture of extensor pollicis longus, and MRI revealed a very rare and aggressive anatomical variant of Lister's tubercle. The patient underwent a transfer of the extensor indicis propius, which is the most popular technique used for hyperextension and retropulsion restitution. Aggressive anatomical forms of Lister's tubercle can explain the frequent occurrence of spontaneous rupture of extensor pollicis longus in patients who practice sports or professions with a repetitive movement of the wrist.

## Introduction

Subcutaneous rupture of the extensor pollicis longus is a very rare injury [1]. Most often, it is a complication of a distal radius fracture, rheumatoid arthritis, and corticosteroid use, but currently, more cases are being reported in patients with a particular profession or practicing certain sports [2]. Surgical treatment is the gold standard, based either on tendon graft or tendon transfer.

## Case presentation

**Figure 1:**
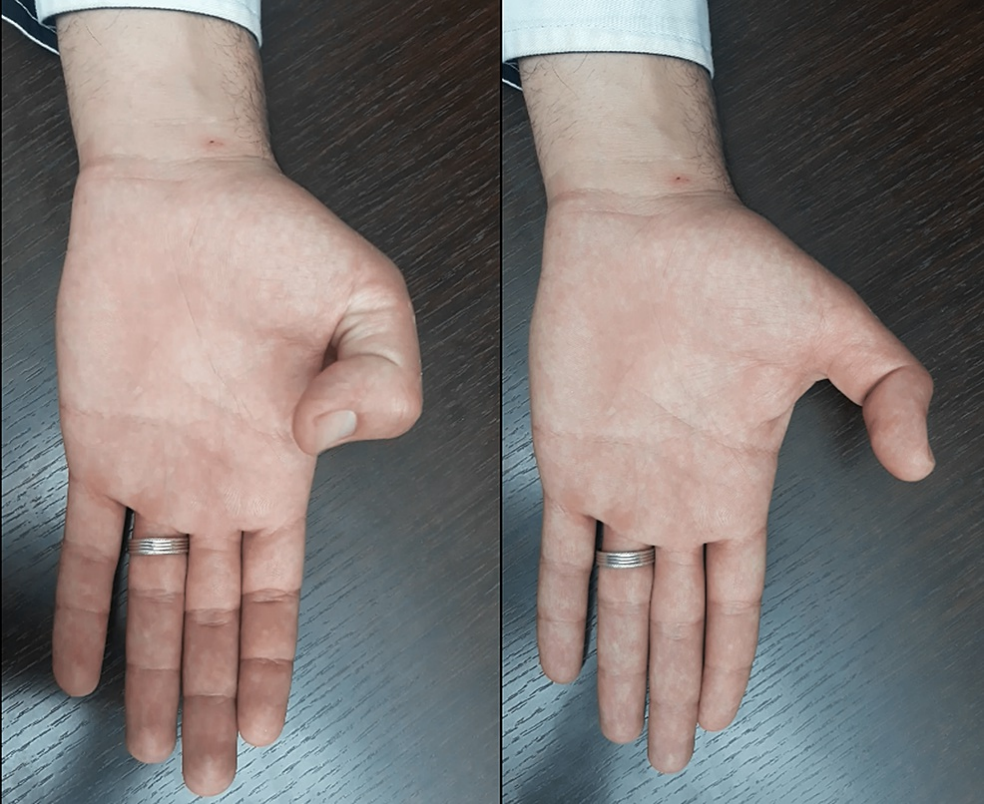
Preoperative image showing mallet thumb and active retropulsion loss

**Figure 2:**
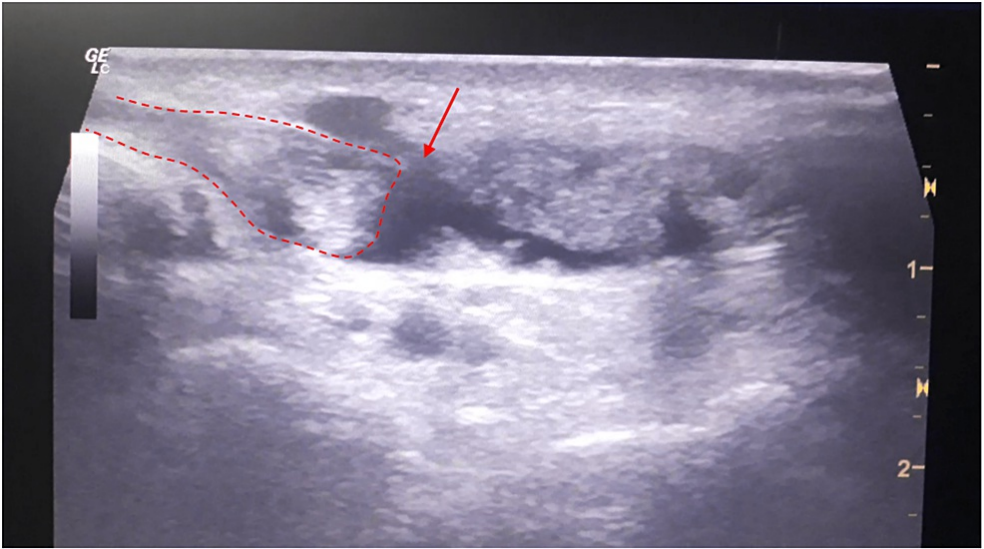
Ultrasound image showing discontinuous extensor pollicis longus in longitudinal scans (red dots) with hematoma (arrow)

**Figure 3:**
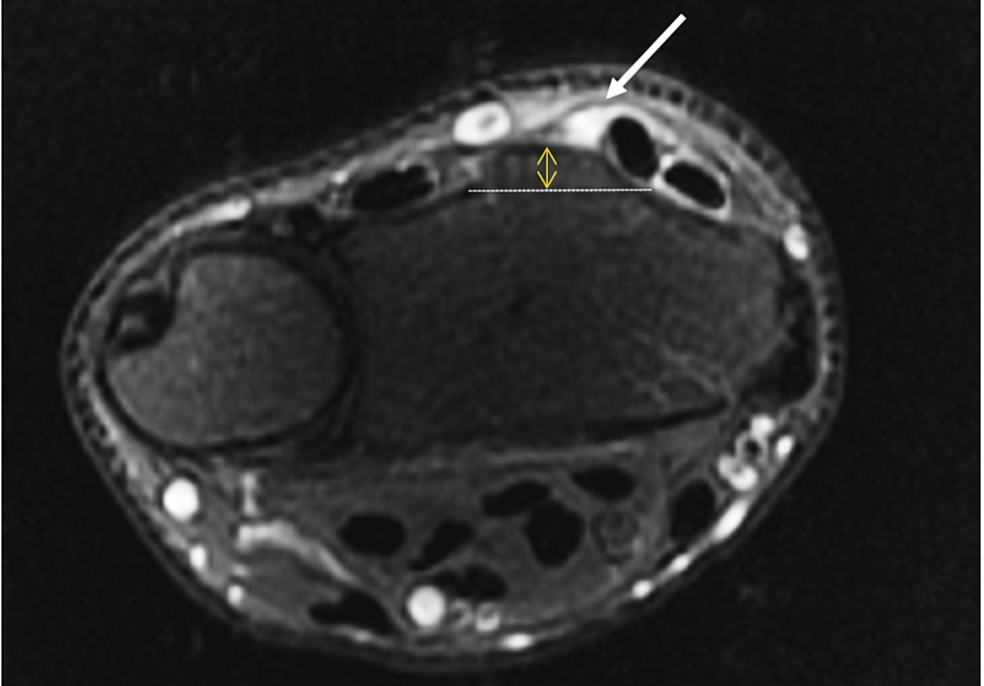
Axial fat-saturation (FAT-SAT) image of the wrist level demonstrating a complete rupture of the extensor pollicis longus (arrow) with no radial peak of Lister's tubercle

**Figure 4:**
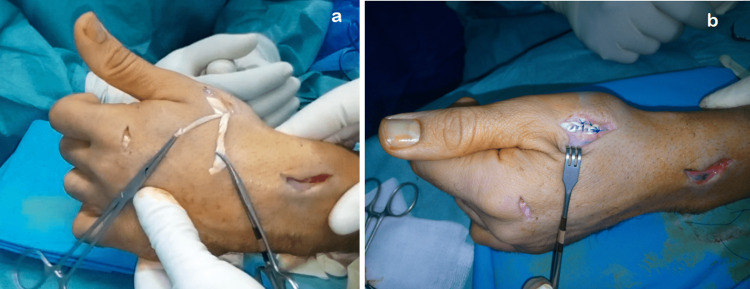
Peroperative images showing (a) the tensioning step before suturing the transferred extensor indicis proprius, (b) the pulvertaft suture of the extensor indicis proprius on the extensor pollicis longus

**Figure 5:**
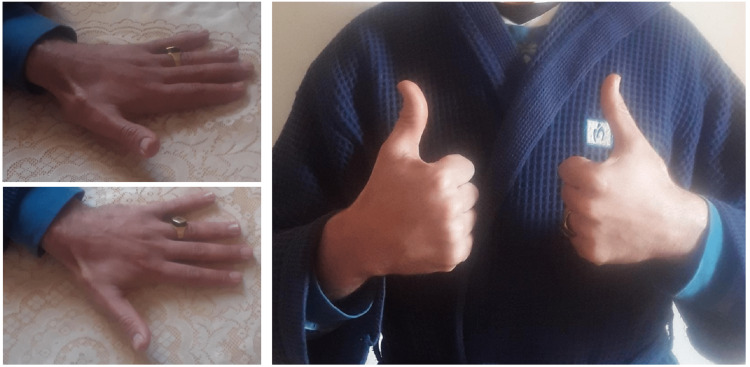
Satisfactory recovery of extension and retropulsion of the thumb (results after three years)

We report the case of a 27-year-old male, a semi-professional bodybuilder with no prior medical history. The patient experienced pain in the left wrist while trying to open a door, followed by an immediate loss of active hyper-extension in the thumb. The patient sought medical attention two weeks later and, upon examination, we found a mallet thumb with a loss of active interphalangeal hyper-extension and retropulsion (Figure 1). Standard radiography did not show any abnormalities. Ultrasound revealed a loss of continuity in the extensor pollicis longus at the wrist level (Figure 2), confirmed by MRI, in association with a 3B form of the Lister's tubercle anatomical variants classification as suggested by Chan et al. (Figure 3), which is the rarest and most aggressive form. The patient underwent transfer of the extensor indicis tendon (Figure 4), resulting in complete recovery of the interphalangeal hyper-extension of the thumb. The three-year evolution is very satisfactory (Figure 5).

## Discussion

Subcutaneous ruptures of the extensor pollicis longus are very rare injuries [1]. Two theories can explain the mechanism of injury. The mechanical theory incriminates friction and tendon path around Lister's tubercle. Then the vascular theory explains that the risk of ischemia at this level is related to the increase in pressure in this inextensible osteofibrous tunnel, exacerbated by the poor vascular irrigation of the tendon in view of Lister's tubercle, making it even more vulnerable [2-4]. The most common etiologies are distal radius fractures, rheumatological disorders, corticosteroid use, and some sports activities (skiers, kick-boxers…) and professions involving repetitive forced wrist movements (cooking, cow milking…) [5-8]. Typically, the patient presents with a loss of active extension of the interphalangeal joint of the thumb for several days or weeks, following sometimes a history of pain at the radial edge of the wrist. The clinical examination finds a thumb in the swan's neck or, more rarely, in mallet deformity, a loss of active interphalangeal joint extension, with the impossibility of the thumb's retropulsion [9]. Ultrasound and MRI can locate the lesion and measure the gap between the tendon edges when possible. Chan et al. proposed a morphological classification in their study of the anatomical variations of Lister's tubercle. By studying 360 wrist MRIs, they have described three anatomical types of Lister's tubercle with two subtypes each. Type 1a is the most common and least aggressive, and 3b is the rarest and the most aggressive [10]. The management of spontaneous ruptures of extensor pollicis longus is always surgical. Two techniques are mostly used. The first is the longus palmaris tendon graft. This technique has the disadvantage of requiring that the graft passes inside the third compartment with a pulvertaft suture further away, to recover the retropulsion and avoid lateral sweeping of the new tendon in the absence of a pulley. The second and most used technique is the extensor indicis proprius transfer. This technique provides excellent results, and it offers the advantage of performing a single suture of a vascularized tendon, providing a course and strength similar to those of the native extensor pollicis longus [11,12].

## Conclusions

The spontaneous rupture of the extensor pollicis longus is a rare injury. Some sports and professional activities requiring repetitive movement of the wrist are increasingly reported in the literature as an etiology of this injury. Lister's tubercle is probably the principal cause. Extensor indicis proprius tendon transfer is a simple technique that leads to excellent results.
